# Acute Limb Ischemia Caused by Dissection following Percutaneous Coronary Intervention Using Right Radial Approach

**DOI:** 10.1155/2022/4846603

**Published:** 2022-10-26

**Authors:** Haris Ahmed, Jonathan Brown, Brooke Walterscheid, Phebe Abraham, Junaid Alam, Nabeel Ahmad, Syed Arman Raza

**Affiliations:** ^1^Department of Internal Medicine, HCA Houston Kingwood/University of Houston College of Medicine, USA; ^2^Department of Cardiology, HCA Houston Kingwood/University of Houston College of Medicine, USA

## Abstract

Iatrogenic aortic dissection is a rare but potentially fatal complication of percutaneous coronary intervention (PCI). Iatrogenic aortic dissection following PCI is rare with most cases reporting dissection originating within the coronary vessels with propagation into the ascending aorta. In this specific case, dissection was without coronary involvement, with dissection extending from the ascending aorta into the descending aorta and iliac vessels. Although PCI via radial approach is associated with fewer vascular complications than with femoral approach, significant adverse outcomes may still occur and require prompt intervention. This case highlights the highly atypical presentation of iatrogenic aortic dissection following cardiac catheterization presenting as acute limb ischemia. In such patients as with ours, immediate surgical intervention is necessary with overall poor prognosis.

## 1. Introduction

Iatrogenic aortic dissection (IAD) secondary to PCI may present variably and without prompt diagnosis and intervention, while outcomes may be unfavorable. Percutaneous coronary intervention via radial approach has been associated with reduced complications and bleeding rates when compared with femoral approach; however, features associated with radial approach may predispose patients to aortic dissection with coronary involvement. We present the rare case of extensive type A aortic dissection extending into the descending aorta causing limb ischemia following right radial heart catheterization.

## 2. Case Presentation

**Figure 1:**
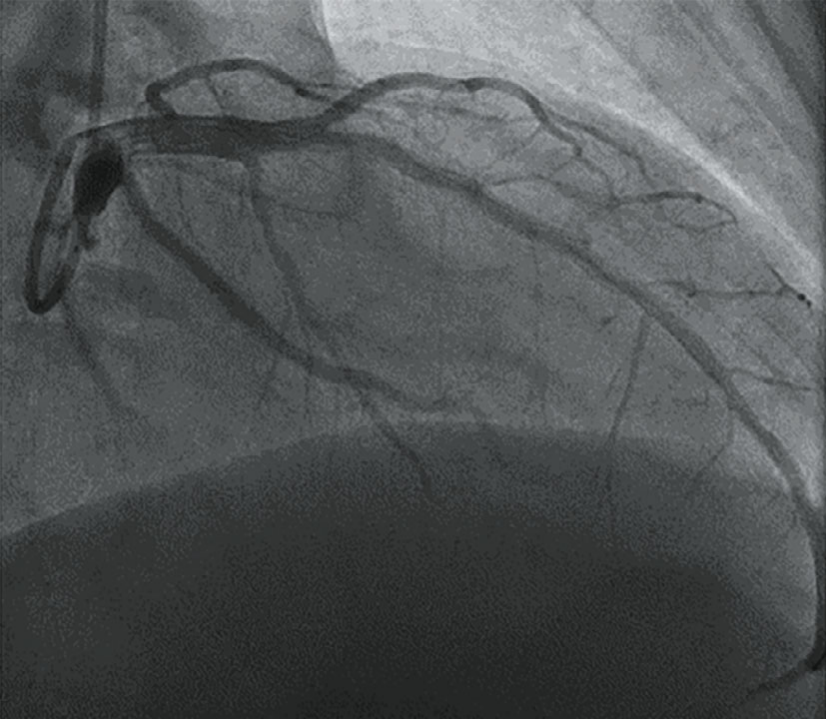
Coronary angiography showing no dissection of the left main coronary artery.

**Figure 2:**
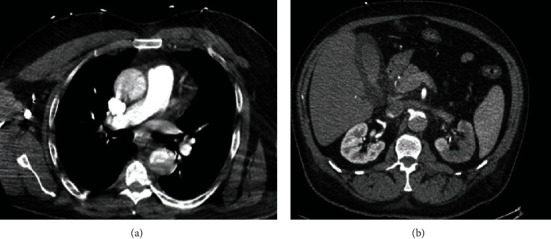
(a) Ascending aortic, descending aortic, and aortic arch aneurysm. (b) L renal infarct and descending aortic aneurysm.

**Figure 3:**
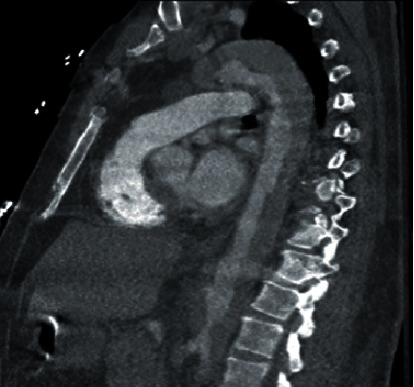
Extensive aortic dissection.

We present a 56-year-old male with past medical history of coronary artery disease status post-PCI ×2 presenting with chest pain and dyspnea. Labs were remarkable for elevated troponin (1.24 ng/mL), and EKG findings were consistent with NSTE-ACS with ST depression more so within inferior leads. He was medically optimized with ACS directed therapy while troponins continued to uptrend (3.04 ng/mL) overnight. Transthoracic echocardiogram demonstrated normal systolic function (EF: 50-54%) with normal aortic root diameter (3.4 cm) and aortic valve function. Right radial arterial area was prepared, and a 5-French Tiger catheter was used to perform the right coronary angiography. Catheter was unsuccessful in engaging the left coronary artery despite multiple attempts and therefore was replaced with a 6-French JL4 diagnostic catheter, and left coronary angiography was performed. Left heart catheterization was remarkable for significant 80% stenosis of the mid left anterior descending artery (LAD). For the PCI part of the procedure, the EBU 4, 6-French guide catheter was used to support and engage the left coronary artery, and the 0.014 runthrough wire was used to cross the lesion and parked distally. The target lesion at the mid LAD was predilated using the 3.0 × 20 mm Emerge compliant balloon with sequential inflation followed by the placement of a Boston Scientific Synergy 3.5 × 48 mm drug-eluting stent from proximal to mid LAD. Post-PCI results were satisfactory with TIMI grade 3 flow and without dissection of left main and other coronary arteries (Figure 1). Within a few hours after the procedure, the patient developed left lower extremity paresthesia with complete loss of pulses and severe left flank pain. Arterial Doppler showed occlusion/stenosis of the left common femoral artery with near absent distal flow. Heparin drip was initiated due to concern for embolic occlusion with subsequent return of pulses, and vascular surgery was consulted. CTA chest and abdominal aorta demonstrated an ascending and descending aortic dissection (Figure 2(a)) extending into (1) left main renal artery with left kidney infarction (Figure 2(b)), (2) inferior mesenteric artery with possible right colon ischemia, and (3) >70% narrowing in the left common iliac artery. CTA chest further reviewed the extensive type A dissection involving the aortic root which had increased in size to 4.6 cm with extension into the right brachiocephalic and right common carotid artery (Figure 3). Upon return from CT, patient was unarousable and was intubated by the ICU team. CT head without contrast did not show intracranial hemorrhage or any acute abnormality. Heparin drip was discontinued, and patient was managed in the intensive care unit with goal of maintaining systolic blood pressure of <120 mmHg. Patient was transferred to a tertiary medical center for an elephant trunk aortic replacement; unfortunately, the patient expired after the intervention.

## 3. Discussion

Radial approach has gained significant interest and usage within interventional cardiology. One of the more significant aims of this approach includes decreasing bleeding and postoperative complications. Comparisons between percutaneous angioplasty using radial and femoral approach showed similar clinical outcomes with decreased access site complications using radial approach [1]. Despite data supporting radial approach, proficiency and experience with this technique plays a pivotal role [2]. Ball et al. showed that success with transradial PCI, comparable to an experienced operator, is dependent on reaching a minimum case volume of ≥50 cases [2]. A lower case volume was associated with increased PCI failure, increased contrast use, and longer fluoroscopy time [2]. Certain limitations with radial approach such as anatomic variation and tortuosity can lead to significant complications [3]. Overall intraoperative aortic dissection (IAD) following PCI is exceedingly rare, as evidenced in a review of 139,262 patients who underwent cardiac catheterization in which the incidence of IAD was 0.06% [4]. Most cases in literature highlight IAD following PCI as a result of retrograde propagation of coronary artery dissection [5]. Interestingly, few cases have reported aortic dissection following heart catheterization without dissection originating within the coronary vessels. In our case, the coronary angiogram postprocedure revealed normal coronary vessels without dissection, pointing to a supracoronary entry point. Upon review of other similar cases, most occurred via right radial access with entry point of dissection occurring around the junction of the right subclavian and brachiocephalic artery. Anatomic anomalies of the subclavian-brachiocephalic axis and tortuosity of the upper limb arteries may predispose such vessels to catheter-induced trauma [6]. The causative factor in the reviewed cases appears multifactorial (calcified vessels, cystic media degeneration, severe atherosclerosis); however, catheter-induced trauma appears to be the focal causative factor. In similar cases, Khan et al. attributed their case to aggressive manipulation of the guide wire into the subclavian loop [3], whereas Noguchi et al. postulated aggressive manipulation of the guiding catheter through the radial artery approach causing intimal tear around the brachiocephalic artery [6]. Similarly, in our case, catheter-induced trauma using right radial approach was the likely inciting factor due to difficulty in engaging the left coronary artery despite multiple attempts.

What makes our patient's case incredibly unique is the extent of dissection from the ascending aorta into the abdominal aorta and run-off vessels affecting the relative organ systems. In this case, the patient's symptoms of severe flank pain and lower extremity paresthesia soon after PCI were the only emergent signs of the underlying complication. Imaging confirmed extensive dissection of the abdominal aorta with dissection extending into the left renal, inferior mesenteric, and left iliac arteries causing the relative symptoms in this case. Such cases involving the ascending and descending aorta have high mortality rates and require extensive surgery known as the “elephant trunk” procedure. Our patient was sent to a tertiary medical center where replacement of the supracoronary ascending aorta with arch repair was followed by treatment of the distal aortic aneurysm. Unfortunately, due to this patient's critical condition and extensive dissection, he expired soon after the initial procedure. To the best of our knowledge, this is the first case reporting ascending aortic dissection involving the abdominal aorta and vessels causing limb ischemia using right radial approach. In conclusion, although radial access may hold certain advantages, rare but fatal complications exist which may be more frequent using radial approach. Such complications may be secondary to complex anatomy of the upper extremity vasculature predisposing these patients to catheter-induced trauma and subsequent dissection with supracoronary entry [3]. In such cases, immediate recognition and medical management is crucial in avoiding extensive dissection, thereby reducing mortality.

## 4. Conclusion

In conclusion, although radial access may have certain advantages, rare however significant complications may exist likely secondary to the complex anatomy of the upper extremity vasculature predisposing such patients to catheter-induced trauma. In such cases, immediate recognition and medical management is crucial in avoiding extensive dissection, thereby reducing mortality. To the best of our knowledge, this is the first case reporting extensive aortic dissection involving the abdominal aorta and run-off vessels causing limb ischemia using right radial approach.

## Data Availability

The data used to support the findings of this study are included within the article and are cited at relevant places within the text as references.

## Abbreviations

PCI: Percutaneous coronary intervention
NSTE-ACS: Non-ST segment elevation-acute coronary syndrome
GDMT: Guideline directed medical therapy
CTA: Computed tomography angiography
ACS: Acute coronary syndrome
LAD: Left anterior descending artery
IAD: Intraoperative aortic dissection
EKG: Electrocardiogram.

## Additional Points

*Learning Objectives*. (i) Recognizing aortic dissection following cardiac catheterization as a complication. (ii) To understand the potential complications of PCI via radial approach. (iii) Immediate recognition and medical management of aortic dissection is crucial in avoiding extensive aortic dissection.
